# *Let-7* miRNA’s Expression Profile and Its Potential Prognostic Role in Uterine Leiomyosarcoma

**DOI:** 10.3390/cells8111452

**Published:** 2019-11-17

**Authors:** Bruna Cristine de Almeida, Laura Gonzalez dos Anjos, Miyuki Uno, Isabela Werneck da Cunha, Fernando Augusto Soares, Glauco Baiocchi, Edmund Chada Baracat, Katia Candido Carvalho

**Affiliations:** 1 Laboratório de Ginecologia Estrutural e Molecular (LIM 58), Disciplina de Ginecologia, Departamento de Obstetricia e Ginecologia, Hospital das Clinicas da Faculdade de Medicina da Universidade de Sao Paulo, HCFMUSP, SP, BR Av. Dr Arnaldo 455, sala 4121, Cerqueira Cesar, São Paulo 05403-010, Brazil; bruc_10@hotmail.com (B.C.d.A.); lauragonzalezanjos@gmail.com (L.G.d.A.); edmund.baracat@hc.fm.usp.br (E.C.B.); 2 Centro de Investigação Translacional em Oncologia (LIM 24), Instituto do Câncer do Estado de São Paulo (CTO/ICESP) Av Dr Arnaldo 251 sala 23 8 andar, São Paulo 01246000, Brazil; miyuki.uno@hc.fm.usp.br; 3 Department of Pathology, Rede D’OR-São Luiz, Rua das Perobas, 344-Jabaquara, São Paulo 04321-120, Brazil; iwerneck0210@gmail.com (I.W.d.C.); fasoares@me.com (F.A.S.); 4 Hospital A C Camargo Cancer Center, SP, BR R. Tamandaré, 753 Liberdade, São Paulo 05403-010, Brazil; 5 National Institute for Science and Technology in Oncogenomics and Therapeutic Innovation, SP, BR R. Tamandaré, 753 Liberdade, São Paulo 05403-010, Brazil; 6 Department of Gynecology Oncology, A.C.Camargo Cancer Center, Rua Prof Antonio Prudente 211, São Paulo 01509-001, Brazil; glauco.baiocchi@accamargo.org.br

**Keywords:** *let-7* family, miRNA, uterine leiomyosarcoma, non-coding RNA

## Abstract

The *lethal-7 (let-7)* family is an important microRNA (miRNA) group that usually exerts functions as a tumor suppressor. We aimed to evaluate the expression profile of *let-7a, let-7b, let-7c, let-7d, let-7e, let-7f, let-7g*, and *let-7i* and to assess their value as prognostic markers in uterine leiomyosarcoma (LMS) patients. The miRNAs expression profile was assessed in 34 LMS and 13 normal myometrium (MM) paraffin-embedded samples. All *let-7* family members showed downregulation in LMS. Our findings showed that patients with *let-7e* downregulation had worse overall survival (OS) and is an independent prognostic factor (hazard ratio [HR] = 2.24). In addition, almost half the patients had distant metastasis. LMS patients with downregulated *let-7b* and *let-7d* had worse disease-free survival (DFS); they are not independent prognostic factors (HR = 2.65). Patients’ ages were associated with *let-7d, let-7e* and *let-7f* (*p* = 0.0160) downregulation. In conclusion, all the *let-7* family members were downregulated in LMS patients, and the greater the loss of expression of these molecules, the greater their relationship with worse prognosis of patients. *Let-7e* expression might influence the OS, while *let-7b* and *le-7d* might influence the DFS. The lowest expression levels of *let-7d, let-7e*, and *let-7f* were associated with the oldest patients. Our findings indicate strong evidence of *let-7’s* role as a potential prognostic biomarker in LMS.

## 1. Introduction

The capacity to regulate multiple target sequences is a relevant feature of microRNAs (miRNAs) that have earned more attention over the last couple of years [1]. miRNAs are a class of small non-coding RNAs ~22 nucleotides long, which are involved in many essential cellular processes [2]. They present little chance of variation or mutation occurrence [1]. An altered expression of miRNAs may result in tumorigenesis. Several neoplasms have been related to the deregulated expression of miRNAs, including gynecological cancers such as ovary, endometrium, cervical, and uterine sarcomas [3,4].

miRNA expression could be disturbed by carcinogenic agents, chemotherapy, and diverse external stimuli, which could impact genetic and epigenetic programs contributing to the heterogeneous biological behaviors of tumors. Importantly, changes in the abundance of miRNAs in tumors may correlate with the clinical and pathological features of patients. Consequently, miRNAs represent novel prognostic biomarkers and promising translational targets in cancer therapy [5].

The *lethal-7 (let-7)* is a major regulator of differentiation, pluripotency, and apoptosis in eukaryotic cells [6]. The *let-7* family is the largest miRNA family and is composed of 10 mature subtypes, including *let-7a, let-7b, let-7c, let-7d, let-7e, let-7f, let-7g, let-7i, miR-98*, and *miR-202*. All members of the *let-7* family contain identical seed sequences and variable stem-loop regions [7,8]. These miRNAs regulate a series of crucial physiological functions, such as growth, development, muscle formation, cell adhesion and homeostasis, and also play a role as a tumor suppressor [7,9]. The regulation of *let-7*’s synthesis has been widely studied due to its relevance in several physiological mechanisms. Many genes, proteins and factors are involved in this process, such as Lin28 and Lin28B, nuclear factor (NF) 90 and NF45 interactions, certain complete regulatory loops that involved NFκB, IL6, IMP1 and c-Myc genes, and DNA methylation [7].

The loss of *let-7* may occur because of the signaling of network perturbations that involve important protein families and lead to accelerated tumor progression in cancer cells [10]. The downregulation of *let-7* is common in many cancer types, and its replacement for normal expression has been found to prevent cancer growth [11]. In uterine tumors, *let-7* family members are negatively regulated in uterine sarcomas [12,13,14,15], but their roles or functions in leiomyosarcoma (LMS) are still not clear. In addition, *let-7* has been shown to have a potential role in cancer signature and prognosis [11].

LMS is the most common uterine sarcoma [16,17], composed of malignant smooth muscle cells with significant cellularity, nuclear atypia, necrosis, high mitotic index, invasion, and metastasis [18]. This tumor is extremely aggressive, exhibits resistance to standard therapy, and has high rates of progression and relapse [16,18]. Signaling pathways and their molecular mechanisms are responsible for malignant LMS transformation processes are still unknown, but some evidence suggests that tumor instability is a result of multiple genetic and epigenetic errors [4,19,20]. miRNAs may play a role in this process by means of regulating gene expression at the post-transcriptional level [4,20]. Therefore, we comprehensively investigated the effects of the gene expression variation of eight members of the *let-7* family in LMS patients, to assess their diagnostic and prognostic values for this tumor.

## 2. Materials and Methods

### 2.1. Patients’ Samples

The present retrospective study was approved by the Research Ethics Committee of the Faculdade de Medicina da Universidade de Sao Paulo—FMUSP (Number 1.517.306) and was conducted in accordance with the Declaration of Helsinki. Formalin-fixed paraffin-embedded (FFPE) tissues from normal myometrium (MM) and LMS were selected for the study’s development. Samples were provided by Anatomic Pathology at the AC Camargo Cancer Center and Gynecology Discipline, Department of Obstetrics and Gynecology, Clinics Hospital, School of Medicine, University of Sao Paulo.

### 2.2. MicroRNA Isolation and Quantitative Real Time-Polymerase Chain Reaction (qRT-PCR) Analysis

Total RNA was extracted using the ReliaPrep™ FFPE Total RNA Miniprep System kit (Promega, Madison, WI, USA) according to the manufacturer’s instructions. After extraction, all samples were quantified by a NanoDrop 2000 spectrophotometer (Thermo Scientific™, Fremont, CA, USA), and the miRNA concentration was confirmed by a fluorometer Qubit^®^ 2.0 (Thermo Fisher Scientific, Waltham, MA, USA), as recommended by the manufacturer. Reverse transcription was performed as described previously by de Almeida et al. [20].

Eight members of the *let-7* family were selected (*let-7a-5p, let-7b-5p, let-7c-5p, let-7d-5p, let-7e-5p, let-7f-5p, let-7g-5p* and *let-7i-5p*), and the quantitative real time-polymerase chain reaction (qRT-PCR) was carried out using a miScript SYBR^®^ Green PCR-kit (Qiagen, Hilden, Germany) with MIHS-109ZA-Qiagen 96 wells plate (Qiagen, Hilden, Germany). Reactions were incubated for 15 min at 95 °C followed by 40 cycles for 15 s at 94 °C, 30 s at 55 °C, and 30 s at 70 °C. The miRNA expressions were normalized using SNORD68 and SNORD95 based on the geNormTM algorithm analysis software version 3.0 (Biogazelle, Zwijnaarde, Belgium) [21]. The MM samples were used as a reference. All data for relative expression were analyzed in the GeneGlobe Data Analysis Center https://geneglobe.qiagen.com/br/analyze/ (Qiagen, Hilden, Germany), in which the qRT-PCR modules transform the threshold cycle (Ct) values to the calculated results for miRNA expression and also via the comparative Ct method by 2^−ΔΔCt^.

For *let-7a*, because of the presently limited detection in the GeneGlobe Data Analysis, the fold expression calculation was performed manually. Calculations used the mean ΔCt values of the test and reference groups. The ΔCt is calculated by subtracting the Ct from each miRNA of the test or reference group by the Ct mean of the normalizing controls (snoRNA- SN) and (ΔCT = Ct ^miRNA (test/reference)^ - mean Ct^SN^). The fold-change was calculated using the 2^−ΔCt (test)^/2^−ΔCt (control)^ model, represented by 2^−ΔΔCt^.

In silico analysis was performed to identify the main genetic interactions network of the *let-7* family strongly related to available genes at https://ccb-web.cs.uni-saarland.de/mirtargetlink/index.php [22].

### 2.3. Statistical Analysis

All statistical analyses were performed using GraphPad Prism 5.0 (GraphPad Software, San Diego, CA, USA) and SPSS version 21 (IBM Corp., Armonk, NY, USA) for Windows. The distribution of continuous data was analyzed by using the Shapiro–Wilk normality test. Expression levels of the eight miRNAs in the MM and LMS groups were analyzed with the Kruskal–Wallis test. Dunn’s multiple comparison post-test was applied to compare the differences among the eight miRNAs in the LMS group. When we compared the miRNA expression between the two groups (MM and LMS), we used the Mann–Whitney U non-parametric test.

The Kaplan–Meier survival curve of the overall 5-year survival (OS) was analyzed with the *log rank* (Mantel Cox) test and multivariate analysis using Cox’s regression analysis via the Cox proportional hazard model. Survival rates were calculated based on the time or period in months. The time of survival was calculated between the date of surgery and the date of death or the date of the last information (follow-up). The disease-free survival (DFS) was calculated from the date of surgery to the date of relapse in months. The Pearson and Spearman’s rank correlation coefficient was used to measures the strength of the linear relationship between two random variables, for the parametric and non-parametric data respectively [23]. A chi-square test and Fisher’s exact test were used to analyze the differences among the frequencies of each variable. The patients’ age was represented by the mean and standard deviation (mean ± SD). Differences were considered statistically significant at *p* < 0.05.

## 3. Results

The expression profile of the *let-7* miRNA family was evaluated in 34 LMS patients, with their ages ranging from 27 to 91 (54.59 ± 15.77). These samples were compared with 13 normal MM patients with ages ranging from 31 to 54 (44.09 ± 6.848) (*p* = 0.4762). Overviews of the clinical and pathological features of LMS patients are described in Table 1.

**Figure 1:**
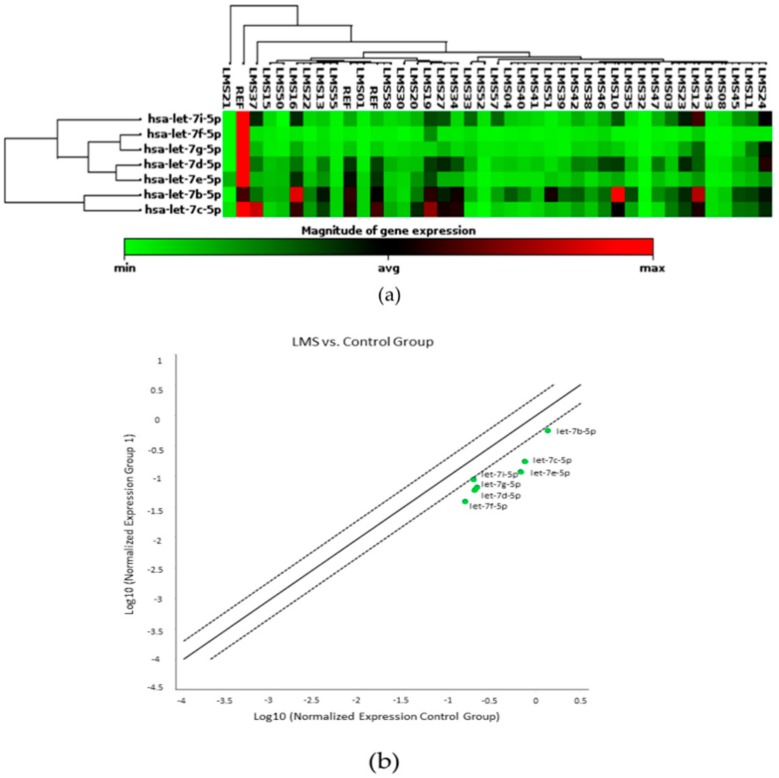
The expression profile of seven *let-7* family members in leiomyosarcoma (LMS), using the myometrium (MM) as a reference (REF) for the normalization of miRNA expression (fold change [FC] expression cut-off values of +2 and –2). (**a**) A clustergram showing the expression of all LMS samples. (**b**) A scatter plot showing the profiling expression of *let-7b, let-7c, let-7d, let-7e, let-7f, let-7g*, and *let-7i*. In green, downregulated miRNAs (Control Group: MM).

Initially, to explore the potential regulatory role of *let-7* miRNAs in LMS, we performed a non-supervised hierarchical clustering analysis of eight members of the *let-7* family (*let-7a, let-7b, let-7c*, *let-7d, let-7e, let-7f, let-7g*, and *let-7i*) in the GeneGlobe Data Analysis Center software. However, *let-7a*’s fold-change in LMS compared to MM could not be calculated by this method even though all quality criteria had been fulfilled. Results show the regulation profiles of these molecules either individually by the LMS sample (clustergram) (Figure 1a) or by group comparison (scatter plots) (Figure 1b). All *let-7* miRNAs showed differential expression with greater downregulation in LMS than in MM. *let-7d* (*p* < 0.0001) and *let-7e* (*p* < 0.0001) showed a highly significant pattern of downregulation. We also observed a statistical significance in the *let-7c* (*p* = 0.0005), *let-7f* (*p* = 0.0002), *let-7g* (*p* = 0.0009), and *let-7i* (*p* = 0.0306) expressions. Only *let-7b* (*p* = 0.0525) did not show a statistical significance in our analysis.

The software could not detect the *let-7a* fold-change values due to the average threshold cycle of this gene which is relatively high (>30) in the control or test sample and is reasonably low in the other sample (<30); then, we performed the fold-change analysis manually as described in the materials and methods section. 

**Figure 2:**
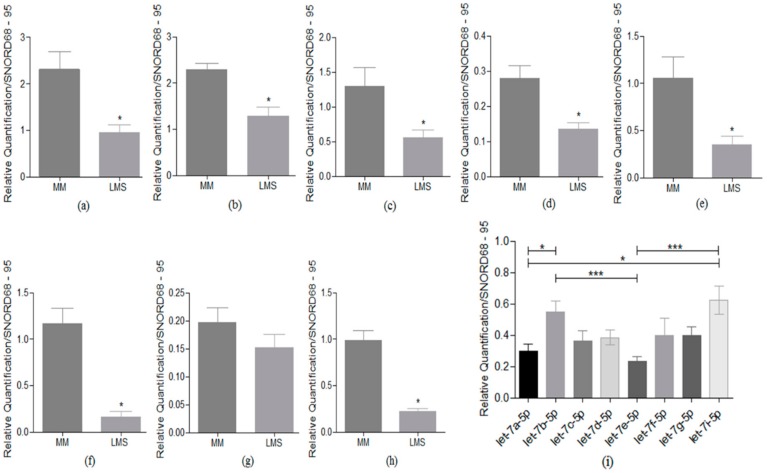
The *let-7* family expression profiles in the LMS and MM samples. (**a**) Differential low expression was observed for *let-7a* (*p* = 0.0280), (**b**) *let-7b* (*p* = 0.0482), (**c**) *let-7c* (*p* = 0.0280), (**d**) *let-7d* (*p* = 0.0221), (**e**) *let-7e* (*p* = 0.0114), (**f**) *let-7f* (*p* = 0.0097), (**g**) *let-7g* (*p* = 0.1367), and (**h**) *let-7i* (*p* = 0.005) in LMS compared to MM, respectively. Samples were normalized endogenously. (**i**) A comparison of the fold expression of the eight members of the *let-7* family in the LMS samples (* *p* < 0.05, ** *p* < 0.005 and *** *p* < 0.0001). Samples were normalized by endogenous and reference samples.

*Let-7a* showed a significantly low expression in LMS compared to MM samples (*p* = 0.0280). We also analyzed the expression profile of *let-7b* (*p* = 0.0482), *let-7c* (*p* = 0.0280), *let-7d* (*p* = 0.0221), *let-7e* (*p* = 0.0114), *let-7f* (*p* = 0.0097), *let-7g* (*p* = 0.1367), and *let-7i* (*p* = 0.005) in LMS compared to MM. *Let-7g* shows no significant differences (Figure 2a–h). The expressions of eight *let-7* miRNAs were compared with the others, to better visualize the differences between these molecules in the LMS. Significant differences were observed between the expressions *let-7a* and *let-7i* (*p* < 0.05) and *let-7b* and *let-7e* (*p* < 0.005). Comparisons between *let-7e* and *let-7i* showed a highly significant expression difference (*p* < 0.0001) (Figure 2i).

The correlation degree between the members of the *let-7* family was evaluated using the Spearman correlation coefficient for age (<50 and ≥50) and relapse (months), but no correlation was found between the variables and the miRNAs expression (data not shown).

As shown in Table 2, we identified the correlation degree of the expression of the *let-7* family. It was found that 10 strong, 6 moderate, and 2 weak positive correlations among *let-7b, let-7d*, and *let-7e* miRNAs and other members of the *let-7* family.

**Figure 3:**
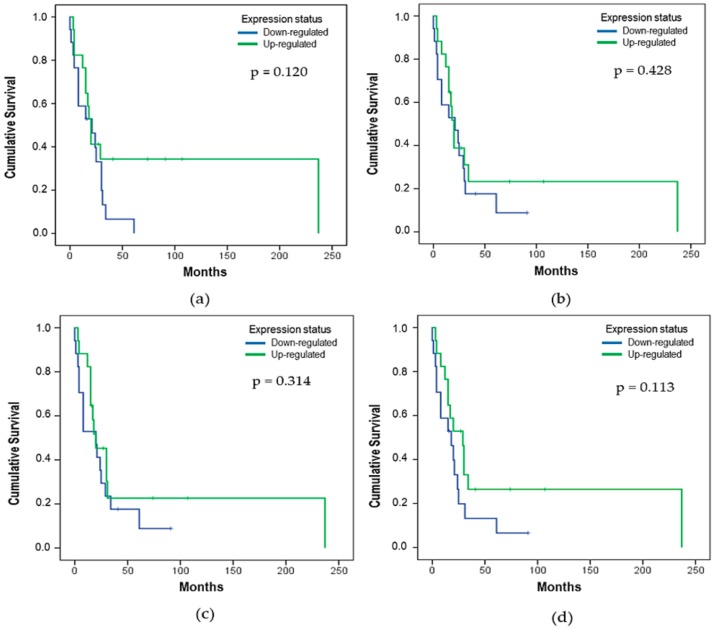
Effect of the expression status on overall survival (OS) for (**a**) *let-7a* (*p* = 0.120), (**b**) *let-7b* (*p* = 0.428), (**c**) *let-7c* (*p* = 0.314), and (**d**) *let-7d* (*p* = 0.113). The p-value was determined using the *log-rank* test that refers to the corresponding expression status (n = 34, 28 deaths, and 6 alive-censored).

**Figure 4:**
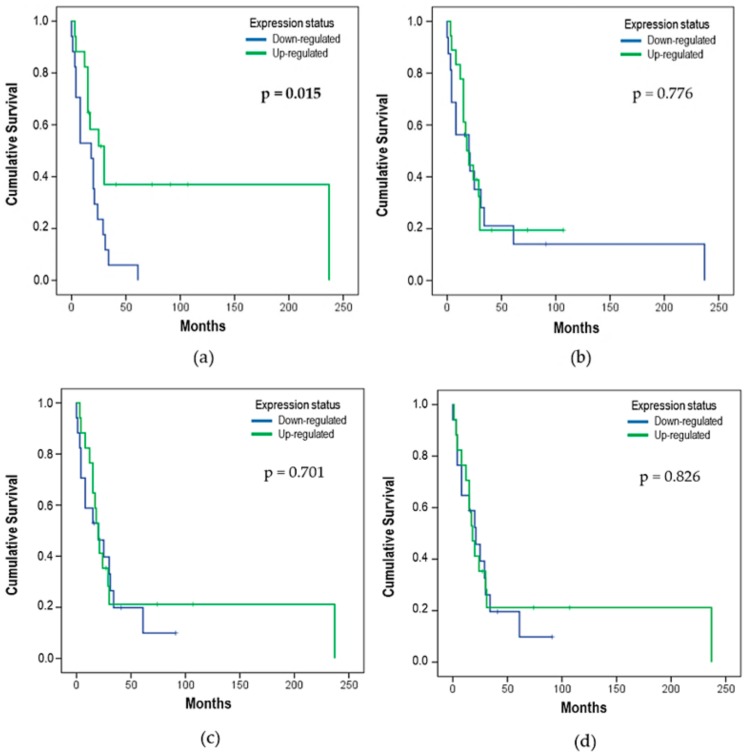
Effect of the expression status on OS for (**a**) *let-7e* (*p* = 0.015), (**b**) *let-7f* (*p* = 0.776), (**c**) *let-7g* (*p* = 0.701), and (**d**) *let-7i* (*p* = 0.826). The p-value was determined using the *log-rank* test that refers to the corresponding expression status (n = 34, 28 deaths, and 6 alive-censored).

The effect of the *let-7* expression status on OS analysis showed that the *let-7e* expression might influence the OS. Patients with downregulated *let-7e* (50%) had a median OS of 18 ± 5.48 months, with 17 deaths (none alive-censored), while patients with upregulated *let-7e* (50%) had a median of 30 ± 7.64 months and 11 deaths (6 alive-censored) (*p* = 0.015). Kaplan–Meier curves were used to compare the time of survival between the two different groups (Figure 3 and Figure 4).

**Figure 5:**
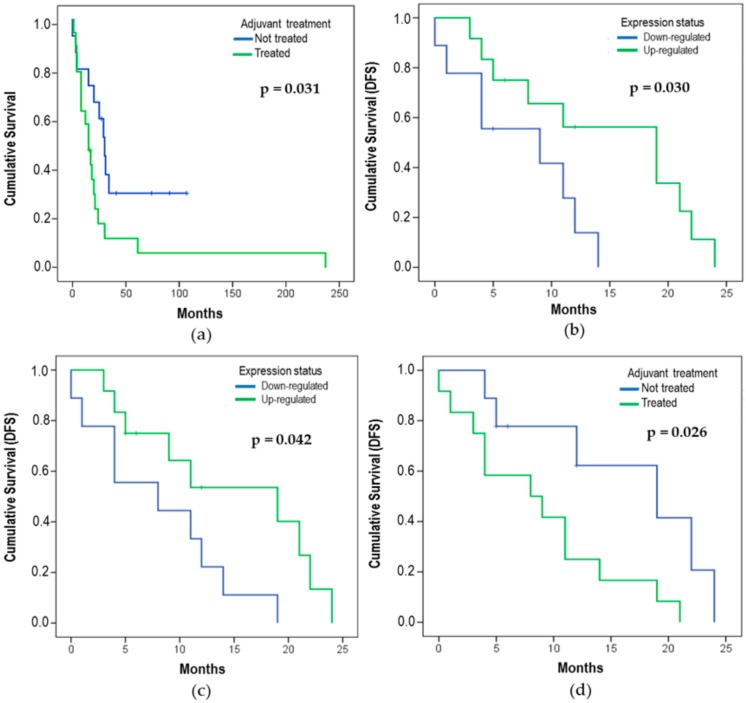
Kaplan–Meier curves showing (**a**) the difference in the time of the OS between treated patients (submitted to adjuvant therapy (AT)–radiotherapy and/or chemotherapy), including 18 cases and non-treated patients with 10 cases (28 deaths and 6 alive-censored) (*log-rank* test: *p* = 0.031). (**b**,**c**) The disease-free survival (DFS) of patients with LMS *let-7b* (*p* = 0.030) and *let-7d* (*p* = 0.042); the p-value was determined using a *log-rank* test that refers to the corresponding expression status (n = 21, 18 relapses, and 3 censored). (**d**) Adjuvant therapy analysis presented 6 relapses for non-treated patients and 12 relapses for treated ones over twenty-five months (18 relapses and 3 censored) (n = 21; *p* = 0.026).

A better survival rate was observed in patients who did not undergo adjuvant therapy (AT) than in those who did the treatment with radiotherapy and/or chemotherapy (*p* = 0.031) in the univariate analysis (Figure 5a).

All variables such as metastasis (*p* = 0.143), relapse (*p* = 0.846), histological grade (*p* = 0.097), and age were subdivided into groups that were <50 and ≥50 years old (*p* = 0.567); these groups were analyzed and did not show any statistical differences (Table 3).

DFS was also evaluated for all *let-7* miRNAs. However, only *let-7b* (*p* = 0.030) and *let-7d* (*p* = 0.042) showed a statistical difference (Figure 5b,c, respectively). *Let-7b* showed that 43% of patients were downregulated (8 relapses and 1 censored) with a DFS median of 9 ± 6.22 months, while 57% were upregulated (10 relapses and 2 censored) with a DFS median of 19 ± 5.40 months. The same analysis was performed for *let-7d*, and the findings demonstrated that 43% of patients were downregulated (8 relapses and 1 censored) with a DFS median of 8 ± 5.96 months, while 57% of patients were upregulated (9 relapses and 3 censored) with a DFS median of 19 ± 6.83 months.

We evaluated the DFS for LMS patients, with or without adjuvant treatment, using a univariate analysis. Our findings showed that treated patients (57%) presented 12 relapses while non-treated patients (43%) had 6 relapses and a better DFS over 25 months (n = 21; *p* = 0.026) (Figure 5d). Some variables did not present statistical differences (Table 4).

Therefore, to understand if the results of *let-7e*’s expression status can influence the AT response in patients who undergo treatment (or not), we proceeded with a multivariate analysis of survival. *Let-7e* expression status proved to be an independent prognostic factor, keeping its statistical difference (Hazard ratio [HR] = 2.24, *p* = 0.048) in the COX regression model (Table 5).

In a further analysis of *let-7b* and *let-7d* expression status, using the Cox regression model, it was found that the expression of both miRNAs was not an independent prognostic factor (Table 5). We observed that patients with LMS and downregulated *let-7b* might have twice as great a risk to develop a disease relapse compared to patients with upregulated *let-7b*. However, in the Cox model, there was no difference (*p* = 0.093).

Regarding patients who underwent AT (n = 19), 68% (13) received chemotherapy, 21% (4) received radiotherapy, and 11% (2) received chemotherapy combined with radiotherapy. Clinical and pathological features were compared between patients treated with adjuvant therapy and those who were non-treated, but no differences were found between the groups. Data not shown here are available in the Supplementary Materials (Table S1).

All clinical and pathological features of LMS patients were exhaustively investigated, taking into account the expression profile of the *let-7* family. Results showed that the loss of *let-7d* (*p* = 0.0158), *let-7e* (*p* = 0.0191), and *let-7f* (*p* = 0.0160) expressions was associated with the oldest patients (Table S2, Supplementary Materials). In addition, 48% of patients who were *let-7e* downregulated had distant metastasis, and 30% of patients with upregulated *let-7e* had local metastasis (*p* = 0.0094). Other variables did not present significant results. Clinical and pathological data not shown here are available in the Supplementary Materials (Tables S3–S10).

**Figure 6:**
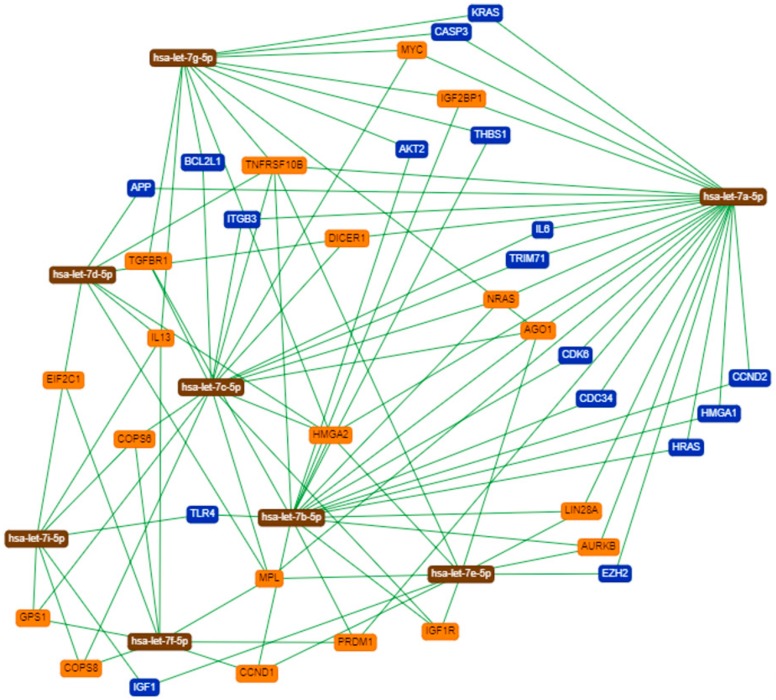
Interaction network of *let-7a-5p, let-7b-5p, let-7c-5p, let-7d-5p, let-7e-5p, let-7f-5p, let-7g-5p and let-7i-5p* with strong support.

**Figure 7:**
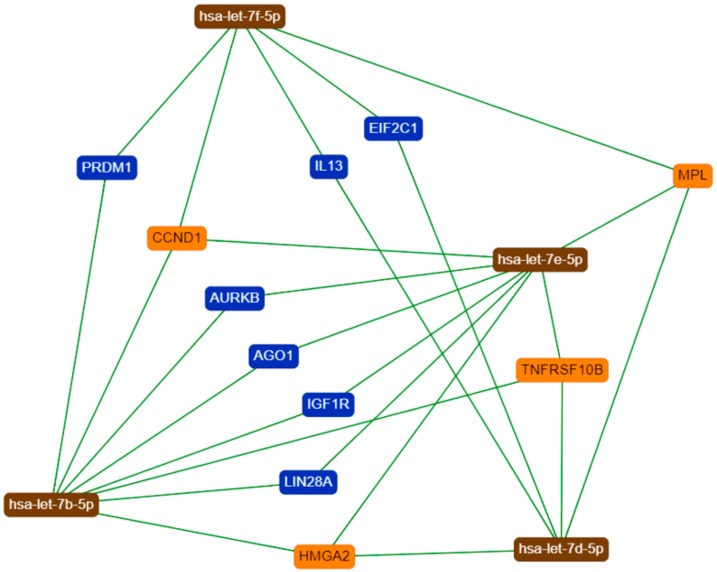
Interaction network of *let-7b-5p, let-7d-5p, let-7e-5p* and *let-7f-5p* and their main target-genes. The network include only regulation with strong evidences defined in the literature.

The network of genetic interactions of let-7 family and their main target-genes is described in Figure 6 and Figure 7.

## 4. Discussion

This retrospective study specifically evaluated the effects and expression profile of eight miRNAs in LMS patients’ clinical outcomes through joint analyses with their clinical and histopathological information.

Cancer-related miRNAs are also called oncomirs due to their ability to act as an oncogene when they are upregulated; they are also a tumor suppressor when they are deleted or downregulated [24]. The *let-7* family is highly conserved, exhibiting an identical seed sequence. Indeed, the differentiation among the members is only 1–4 nucleotides. These congeners share common target mRNA [9]. The role of the *let-7* family as a tumor suppressor is well characterized for multiple cancers [25]. Deregulation at the expression level of these molecules has also been observed in pancreatic cancers, as well as prostate cancer, primary pigmented nodular adrenocortical disease (PPNAD), head and neck malignancies, and ovary, breast, bladder, kidney and retinoblastomas [26]. Downregulation was found for cervical cancer [27], epithelial mesenchymal tumors [15], and endometrial stromal sarcomas [4,15]. Our group previously found evidence of let-7′s loss of expression in uterine LMS [4] and the uterine LMS cell line [20]. The findings of the two earlier studies and the current data are in line with Shi et al. [28], and Kowalewska et al., [15]. Even though they used a less expressive number samples.

The non-supervised hierarchical clustering analysis showed that there is a marked loss of expression for all the *let-7* family members evaluated in the LMS relative to the reference tissue MM. Although *let-7a*’s fold-change was not observed in this analysis, we showed (specifically in Figure 2a) that this molecule has the same profile as the other members, since it also showed significantly low expression in the LMS compared to benign tissue.

In the second analysis, we used the fold regulation cut-off values of +3 and −3 and observed that, specifically, *let-7d* and *let-7e* featured the most expressive differences between LMS and MM (data not shown). According to the literature, the function and expression of *let-7e* vary in different types of tumors. *Let-7e* has been demonstrated to act as a tumor suppressor via its downregulation in colorectal, esophagus, and ovarian cancers, as well as in lymphoma and non-small cell lung carcinoma (NSCLC). In contrast, there is an increase of *let-7e* expression in retinoblastoma and synovial sarcoma. These results suggest that *let-7e* exerts a tumor-specific role [29]. Although *let-7d* has a high transcription rate, it seems to be less studied than other members of its family. It has also been observed that *let-7d* can perform anti-oncogenic and oncogenic roles [30]. Despite the wide sequence similarity among the molecules, Lee et al. [31] described that *let-7d* and *let-7e* have a shorter nucleotide than other members of the family. This peculiarity, together with the in silico analysis that shows the similarities between *HMGA2* and other target genes [32], indicate a possible cooperation in the regulation of gene expression. 

For a better understanding of the interaction among the miRNAs of the *let-7* family, we performed a comparative analysis. Although a similar profile was characterized, there was a significant difference in the expression between some members, with the most representative occurring in *let-7e* and *let-7i*.

In humans, *let-7g* and *let-7i* are individually located on chromosomes 3 and 12, respectively, while the other members of the *let-7* family are distributed among four groups (cluster 1 to 4). *Let-7e* is inserted into Cluster1-c, which comprises *let-7e, miR-99b,* and *miR-125a*, located on chromosome 19 [33]. Gene amplification or deletion, abnormalities in the transcriptional control of miRNAs, epigenetic changes, and disorders of the mechanism of biogenesis are the factors responsible for the alteration in miRNA levels in human cancers [34]. Considering that mutational profiles are specific to each tumor, these differences may be important for the definition of a unique molecular signature in the LMS.

Correlation analyses showed that members of the *let-7* family tend to lose their expression together. Two highly conserved binding proteins, LIN28A and LIN28B, may be involved in this process because of their ability to inhibit *let-7* biogenesis in mammals by directly binding to the pre-let-7 processed by Dicer and/or pri-let-7 processed by Drosha. Thus, it is believed that *LIN28* acts as an oncogene, at least in part because of its role in suppressing *let-7* family members. High levels of *LIN28A* or *28B* are found in many cancers, such as glioblastoma, ovarian, stomach, prostate, and breast cancer [35,36]. Due to the important role of the *LIN28*/*let-7* axis in developmental biology and cancer, the mechanisms underlying the post-transcriptional suppression of *let-7* miRNAs by *LIN28* have been intensively investigated [2,37]. Previous studies suggest that the *LIN28/let-7/c-MYC* pathway played a significant role in the development and progression of several cancers types [37].

Another regulation mechanism for miRNAs maturation occurs through Dicer recruitment. This RNase III endonuclease is aberrantly expressed in different types of cancer and it has been reported to be regulated by the *let-7* family. The Dicer showed high expression levels correlated with a lower expression of *let-7b* leading to increased cell proliferation in oral cancer cells [38]. The Fas (also termed APO-1 or CD95) is also included in the regulation process and it has been reported to be regulated by *let-7/miR-98* in T cells. This protein when is activated inhibits Dicer, reducing the levels of mature *let-7* miRNA [7]. In addition, Brueckner et al. [39], observed that the human *let-7a-3* gene on chromosome 22q13.31 was associated with a CpG island, being hypermethylated in normal human tissues, but the gene was hypomethylated in some lung cancers. The *let-7a-3* was identified as a miRNA gene that is epigenetically regulated, suggesting that aberrant methylation might contribute to the human cancer epigenome.

The association of an unfavorable prognosis in cancer patients with the downregulation of *let-7* family members is already well described in the literature. Downregulation was observed in the *let-7* (more specifically, *let-7a-2*) and correlates with poor survival in lung cancer, while the loss of the expression of *let-7d* in head and neck squamous cell carcinoma and ovarian cancer was also indicative of poor survival [40]. 

Notably, our results show that downregulation was also a worse prognostic factor in patients with LMS. We observed that patients who presented *let-7e* and *let-7b* downregulation had twice the risk of death and a greater risk of relapse, respectively. Wu et al. [41] speculated that the total dose of *let-7* is progressively determined by regulating the levels of each individual member, which are maintained at a level that can suppress the cancer’s development. This study emphasizes that distinct levels of these molecules mediate favorable phenotypes.

We also found that patients who did not undergo AT had a better disease outcome with a favorable DFS. Evidence shows that miRNAs play a relevant role in multidrug resistance through abnormal modulation of ATP-binding cassette (ABC) transporter genes, apoptosis-related genes, and autophagy, drug metabolism genes, and redox systems [42]. In addition to the absence of adjuvant therapeutic regimens that improve survival in this population [43], Pautier et al. [44] described the high cytotoxicity of chemotherapy performed on patients with uterine sarcomas. The authors attributed the occurrence of two deaths to the side effects of treatment. Other studies noted that both adjuvant chemotherapy and radiotherapy are not associated with a significant survival benefit and cannot be considered the standard methods for uterine LMS [45,46]. However, it is important to consider the sample size and patient heterogeneity. More well-controlled studies are necessary to identify or establish the real effects of AT on the OS in LMS patients.

Curiously, we also verified an association between older patients and the downregulation of *let-7d, let-7e* and *let-7f* miRNAs. The aging process is related to characteristics such as genomic instability, telomeric attrition, epigenetic alterations, loss of proteostasis (protein homeostasis), nutritional imbalance, mitochondrial dysfunction, cellular senescence, stem cell exhaustion, and altered intercellular communication [47]. There is an increase in the incidence of LMS cases in women over 50 years old [48], and strong evidence shows that miRNAs play an important role in modulating the life span and aging process in these women. Distant metastasis was another factor associated with *let-7* expression—more specifically, *let-7e*. Ma et al. [49] identified that the previously mentioned *miR-99b/let-7e/miR-125a* cluster acts as a pro-metastatic agent in esophageal squamous cell carcinoma. This cluster also demonstrates overexpression in multiple myeloma, synovial sarcoma, and colorectal cancer. Despite these recent findings, the specific function of this cluster remains unknown in LMS and other cancers.

We expanded our investigations to available databases, such as The Cancer Genome Atlas (TCGA) [50], Cbioportal [51], and Cosmic [52]. We found that *let-7* data are available for uterine carcinomas (CS) and endometrioid endometrial cancer (EEC), but information still needs to be added for LMS. The genetic interactions network of let-7′s with their main target-genes were performed using miRTargetLink Human database [22]. Some target-genes identified are well described in the literature with an association in LMS development. However, these findings open a new perspective for further studies, since the role of many potential genes is still unknown.

LMS are highly aggressive, heterogeneous tumors with a largely unknown molecular basis. The optimization and expansion of the therapeutic options for these tumors remain an unsolved clinical need [53]. Our results, although based on a limited number of samples, indicate that the *let-7* family may be a potential prognostic biomarker and may assist in the development of molecularly targeted drugs.

## 5. Conclusions

In conclusion, our findings showed that all *let-7* family members were downregulated in LMS, apparently acting as a tumor suppressor in smooth muscle tissue. Almost half of the patients with a downregulation of *let-7e* had distant metastases and a lower overall 5-year survival. The downregulation of *let-7e* may increase the death risk for LMS’ patients and seems to be an independent prognostic factor.

In the DFS, *let-7b* downregulation was related to a greater rate of relapse. Patients who did not undergo adjuvant therapy had a better DFS, and their *let-7b* and *let-7d* expression status appears not to be an independent prognostic factor. Moreover, patients with *let-7b* downregulation are at twice the risk of relapsing, indicating that downregulation is associated with a worse prognosis. In addition, the downregulation of *let-7d*, *let-7e*, and *let-7f* was associated with the oldest patients.

## Figures and Tables

**Table 1:** Clinical and histopathological features of leiomyosarcoma patients (n = 34).

Variables	Overall Population	Percentage Frequencies
Clinical FIGO stage		
I	13	38%
II	5	15%
III	6	18%
IV	10	29%
Relapse		
No	11	32%
Yes	23	68%
Metastasis		
No	2	6%
Local	10	29%
Distant	19	56%
Local and distant	3	9%
Menopause *		
Yes	8	26%
No	23	74%

* Data missing (n = 3). FIGO, International Federation of Gynecology and Obstetrics.

**Table 2:** Correlation degree of the expression of the *let-7* family.

*let-7* Family Member		Correlation Coefficient (r)	*p*
***let-7b***	*let-7a*	0.7283	**<0.0001**
	*let-7c*	0.7678	
	*let-7d*	0.7901	
	*let-7g*	0.7213	
	*let-7e*	0.6321	
	*let-7f*	0.6113	
	*let-7i*	0.6700	
***let-7d***	*let-7a*	0.7488	**<0.0001**
	*let-7c*	0.8478	
	*let-7f*	0.7675	
	*let-7g*	0.8952	
	*let-7i*	0.7766	
	*let-7e*	0.6865	
***let-7e***	*let-7c*	0.7806	**<0.0001**
	*let-7a*	0.6684	**<0.0001**
	*let-7g*	0.6263	**<0.0001**
	*let-7f*	0.5917	**0.0002**
	*let-7i*	0.5013	**0.0025**

**Table 3:** Univariate analysis for overall survival.

Variable	Median ± SE	95% CI	*p*
Age (n = 34)			
<50	20.0 ± 6.84	6.58–33.41	0.567
≥50	18.0 ± 5.59	7.04–28.95	
FIGO stage (n = 34)			
I and II	25.0 ± 5.33	14.54–35.45	0.142
III and IV	15.0 ± 2.97	9.16–20.83	
Adjuvant therapy (n = 34)			
No	30.0 ± 4.40	21.37–38.62	0.031
Yes	15.0 ± 3.48	8.17–21.82	
Histologic grade (n = 34)			
Low-grade	31.0 ± 10.0	11.40–50.60	0.097
High-grade	17.0 ± 2.92	11.27–22.72	
Relapse (n = 27)			
No	61.0	-	0.078
Yes	20.0 ± 2.39	15.30–24.69	
Metastasis (n = 34)			
No	61.0	-	0.143
Yes	18.0 ± 3.36	11.41–24.58	

SE, standard error; CI, confidence interval; FIGO, International Federation of Gynecology and Obstetrics.

**Table 4:** Univariate analysis for the DFS/Kaplan–Meier test (n = 21).

Variable	Median ± SE	95% CI	*p*
Age			
<50	9.0 ± 4.78	0.00–18.37	0.758
≥50	11.0 ± 2.25	6.58–15.42	
FIGO stage			
I and II	12.0 ± 4.52	3.14–20.85	0.834
III and IV	11.0 ± 1.54	7.96–14.03	
Adjuvant therapy			
No	19.0 ± 6.97	5.32–32.67	0.026
Yes	8.0 ± 4.33	0.00–16.48	
Histologic grade			
Low-grade	14.0 ± 00.0	-	0.642
High-grade	11.0 ± 1.97	7.12–14.87	

SE, standard error; CI, confidence interval; DFS, disease-free survival; FIGO, International Federation of Gynecology and Obstetrics.

**Table 5:** Multivariate proportional hazard analysis (Cox model) of the adjuvant therapy status and expression of miRNAs.

Variable		HR (95% CI)	*p*
OS	*let-7e* expression ^1^	2.24 (1.00–5.00)	0.048
	Adjuvant therapy ^2^	0.495 (0.21–1.12)	0.092
DFS	*let-7b* expression ^1^	2.65 (0.84–8.29)	0.093
	Adjuvant therapy ^2^	0.35 (0.11–1.12)	0.079
	*let-7d* expression ^1^	1.87 (0.61–5.77)	0.271
	Adjuvant therapy ^2^	0.39 (0.11–1.35)	0.139

^1^ Compared to greater expression; ^2^ Compared to treatment. OS, overall survival; DFS, disease-free survival; HR, hazard ratio; CI, confidence interval.

## Supplementary Materials

The following are available online at https://www.mdpi.com/2073-4409/8/11/1452/s1, Table S1: Analysis of clinical and pathological features of patients treated and untreated with adjuvant therapy. Table S2: Patient age analysis according to the miRNA expression profile. Table S3: Comparison between clinical and pathological features and *let-7a* expression. Table S4: Comparison between clinical and pathological features and *let-7b* expression. Table S5: Comparison between clinical and pathological features and *let-7c* expression. Table S6: Comparison between clinical and pathological features and *let-7d* expression. Table S7: Comparison between clinical and pathological features and *let-7e* expression. Table S8: Comparison between clinical and pathological features and *let-7f* expression. Table S9: Comparison between clinical and pathological features and *let-7g* expression. Table S10: Comparison between clinical and pathological features and *let-7i* expression.
