# Supplementary material for: Let-7 miRNA’s Expression Profile and Its Potential Prognostic Role in Uterine Leiomyosarcoma
Source: Cells. 2019 Nov 17;8(11):1452. doi: 10.3390/cells8111452 (PMC6912804; doi:10.3390/cells8111452)
Supplement: Supplementary file 1 [file cells-08-01452-s001.pdf]

Supplementary Materials

Let-7 miRNA's Expression Profile and Its Potential Prognostic Role in Uterine Leiomyosarcoma

Bruna Cristine de Almeida [1], Laura Gonzalez dos Anjos [1], Miyuki Uno [2], Isabela Werneck da Cunha [3,4,5], Fernando Augusto Soares [3,4,5], Glauco Baiocchi [6], Edmund Chada Baracat [1] and Katia Candido Carvalho [1,*]

[1] Laboratório de Ginecologia Estrutural e Molecular (LIM 58), Disciplina de Ginecologia, Departamento de Obstetricia e Ginecologia, Hospital das Clinicas da Faculdade de Medicina da Universidade de Sao Paulo, HCFMUSP, SP, BR Av. Dr Arnaldo 455, sala 4121, Cerqueira Cesar, São Paulo 05403-010, SP - Brazil; bruc_10@hotmail.com (B.C.A.); lauragonzalezanjos@gmail.com (L.G.d.A.); edmund.baracat@hc.fm.usp.br (E.C.B.)

[2] Centro de Investigação Translacional em Oncologia (LIM 24), Instituto do Câncer do Estado de São Paulo (CTO/ICESP) Av Dr Arnaldo 251 sala 23 8 andar; miyuki.uno@hc.fm.usp.br

[3] Department of Pathology, Rede D'OR-São Luiz, Rua das Perobas, 344-Jabaquara, São Paulo 04321-120, Brazil

[4] Hospital A C Camargo Cancer Center, SP, BR R. Tamandaré, 753 Liberdade, São Paulo 05403-010, Brazil; fasoares@me.com (F.A.S.); iwerneck0210@gmail.com (I.W.d.C.)

[5] National Institute for Science and Technology in Oncogenomics and Therapeutic Innovation, SP, BR R. Tamandaré, 753 Liberdade, São Paulo 05403-010, Brazil

[6] Department of Gynecology Oncology, A.C.Camargo Cancer Center, Rua Prof Antonio Prudente 211, São Paulo, Brazil; glauco.baiocchi@accamargo.org.br

* Correspondence: carvalhokc@gmail.com; Tel.: +55-011-3061-7486

Table S1. Analysis of clinical and pathological features of patients treated with adjuvant therapy and untreated.

Variables	Adjuvant Therapy (n = 19)	No Adjuvant Therapy (n = 15)	*p*
Clinical FIGO stage			0.1907 [a]
1	5 (26%)	8 (53%)	
2	2 (11%)	3 (20%)	
3	5 (26%)	1 (7%)	
4	7 (37%)	3 (20%)	
Relapse			0.9135 [b]
No	6 (32%)	5 (33%)	
Yes	13 (68%)	10 (67%)	
Mitotic count (mitoses/10 high-power fields)			0.2421 [b]
≤10	0 (0%)	3 (%)	
≥10	8 (%)	9 (%)	
Metastasis			0.8622 [a]
No	1 (5%)	1 (7%)	
Local	6 (32%)	4 (27%)	
Distant	11 (58%)	8 (53%)	
Local e distant	1 (5%)	2 (13%)	
Menopause*			0.6980 [b]
Yes	5 (29%)	3 (21%)	
No	12 (71%)	11 (79%)	

* Patients treated with adjuvant therapy n = 17 and non-treated with adjuvant therapy n = 14; [a] Chi-square; [b] Fisher exact. FIGO, International Federation of Gynecology and Obstetrics.

Table S2. Patients ages analysis according to the miRNAs expression profile.

Age (years) n = 34	Downregulated Mean ± SD (min – max)	Upregulated Mean ± SD (min – max)	p*
let-7a	56.35 ± 18.24 (31 – 91)	52.82 ± 13.18 (27 – 75)	0.4641
let-7b	59.35 ± 16.83 (31 – 91)	49.82 ± 13.48 (27 – 75)	0.1048
let-7c	59.12 ± 15.69 (33 – 91)	50.06 ± 14.94 (27 – 84)	0.1335
let-7d	61.47 ± 16.34 (31 – 91)	47.71 ± 12.06 (27 – 72)	0.0158
let-7e	60.94 ± 16.33 (33 – 91)	48.24 ± 12.67 (27 – 72)	0.0191
let-7f	61.63 ± 15.37 (40 – 91)	48.33 ± 13.66 (27 – 78)	0.0160
let-7g	57.47 ± 17.49 (31 – 91)	51.71 ± 13.77 (27 – 78)	0.2242
let-7i	56.24 ± 16.10 (31 – 91)	52.94 ± 15.76 (27 – 84)	0.5349

* *t* teste.

Table S3. Comparison between clinical and pathological features and the *let-7a* expression.

Variables	n (%)	*let-7a*		p
		Downregulated	Upregulated	
**Age (years)**				
<50	34 (100)	7 (21)	7 (21)	1.000 [a]
≥50		10 (29)	10 (29)	
**Menopause**	31 (100)			
No		5 (16)	3 (10)	0.4331 [a]
Yes		10 (32)	13 (42)	
**Histologic grade**	34 (100)			
Low grade		3 (9)	3 (9)	1.000 [b]
High grade		14 (41)	14 (41)	
**Clinical FIGO stage**	34 (100)			0.4675[a]
I		8 (21)	5 (18)	
II		2 (6)	3 (9)	
III		3 (9)	3 (9)	
IV		4 (12)	6 (18)	
**Adjuvant therapy**	34 (100)			0.6421 [a]
No		6 (18)	9 (25)	
Chemotherapy		7 (21)	6 (18)	
Radiotherapy		3 (9)	1 (3)	
Chemo and Radio		1 (3)	1 (3)	
**Relapse**	34 (100)			0.4646 [b]
No		7 (21)	4 (11)	
Yes		10 (30)	13 (38)	
**Mitotic count (mitoses/10 high-power fields)**	20 (100)			1.000 [b]
≤10		1 (5)	2 (10)	
≥10		9 (45)	8 (40)	
**Metastasis**	34 (100)			1.000 [b]
No		1 (3)	1 (3)	
Yes		16 (47)	16 (47)	
**Metastasis Localization**	26 (100)			0.2016 [b]
Local (pelvic)		2 (8)	6 (23)	
Distant		11 (42)	7 (27)	

[a] Chi-square; [b] Fisher exact; FIGO, International Federation of Gynecology and Obstetrics.

Table S4. Comparison between clinical and pathological features and the *let-7b* expression.

Variables	n (%)	*let-7b*		*p*
		Downregulated	Upregulated	
**Age (years)**				
<50	**34 (100)**	6 (18)	8 (24)	0.1048 [a]
≥50		11 (32)	9 (26)	
**Menopause**	31 (100)			
No		5 (16)	3 (10)	0.4125 [b]
Yes		9 (29)	14 (45)	
**Histologic grade**	34 (100)			
Low grade		4 (12)	2 (6)	0.6562 [b]
High grade		13 (38)	15 (44)	
**Clinical FIGO stage**	34 (100)			0.7310 [a]
I		9 (26)	4 (12)	
II		2 (6)	3 (9)	
III		3 (9)	3 (9)	
IV		3 (9)	7 (20)	
**Adjuvant therapy**	34 (100)			0.6239 [a]
No		8 (23)	7 (21)	
Chemotherapy		5 (15)	8 (23)	
Radiotherapy		3 (9)	1 (3)	
Chemo and Radio		1 (3)	1 (3)	
**Relapse**	34 (100)			1.000 [b]
No		6 (18)	5 (15)	
Yes		11 (32)	12 (35)	
**Mitotic count (mitoses/10 high-power fields)**	20 (100)			1.000 [b]
≤10		1 (5)	2 (10)	
≥10		6 (30)	11 (55)	
**Metastasis**	34 (100)			0.4848 [b]
No		2 (6)	0 (0)	
Yes		15 (44)	17 (50)	
**Metastasis Localization**	23 (100)			0.1930 [b]
Local (pelvic)		2 (9)	6 (26)	
Distant		9 (39)	6 (26)	

[a] Chi-square; [b] Fisher exact; FIGO, International Federation of Gynecology and Obstetrics.

Table S5. Comparison between clinical and pathological features and the *let-7c* expression.

Variables	n (%)	*let-7c*		*p*
		Downregulated	Upregulated	
**Age (years)**				
<50	**34 (100)**	6 (18)	8 (24)	0.7283 [a]
≥50		11 (32)	9 (26)	
**Menopause**	31 (100)			
No		4 (13)	4 (13)	1.000 [b]
Yes		11 (36)	12 (38)	
**Histologic grade**	34 (100)			
Low grade		3 (9)	3 (9)	1.000 [b]
High grade		14 (41)	14 (41)	
**Clinical FIGO stage**	34 (100)			0.4767 [a]
I		8 (23)	5 (14)	
II		3 (9)	2 (6)	
III		3 (9)	3 (9)	
IV		3 (9)	7 (21)	
**Adjuvant therapy**	34 (100)			0.1725 [a]
No		8 (23)	7 (21)	
Chemotherapy		4 (12)	9 (26)	
Radiotherapy		3 (9)	1 (3)	
Chemo and Radio		2 (6)	0 (0)	
**Relapse**	34 (100)			1.000 [b]
No		5 (14)	6 (18)	
Yes		12 (35)	11 (33)	
**Mitotic count (mitoses/10 high-power fields)**	20 (100)			0.5211 [b]
≤10		0 (0)	3 (15)	
≥10		7 (35)	10 (50)	
**Metastasis**	34 (100)			0.4848 [b]
No		2 (6)	0 (0)	
Yes		15 (44)	17 (50)	
**Metastasis Localization**	23 (100)			0.8994 [b]
Local (pelvic)		2 (9)	6 (26)	
Distant		10 (43)	5 (22)	

[a] Chi-square; [b] Fisher exact; FIGO, International Federation of Gynecology and Obstetrics.

Table S6. Comparison between clinical and pathological features and the *let-7d* expression.

Variables	n (%)	*let-7d*		*p*
		Downregulated	Upregulated	
**Age (years)**				
<50	**34 (100)**	5 (15)	9 (26)	0.2960 [b]
≥50		12 (35)	8 (24)	
**Menopause**	31 (100)			
No		5 (16)	3 (10)	0.4331 [b]
Yes		10 (32)	13 (42)	
**Histologic grade**	34 (100)			
Low grade		4 (12)	2 (6)	0.6562 [b]
High grade		13 (38)	15 (44)	
**Clinical FIGO stage**	34 (100)			0.3238 [a]
I		7 (21)	6 (17)	
II		4 (11)	1 (3)	
III		3 (9)	3 (9)	
IV		3 (9)	7 (21)	
**Adjuvant therapy**	34 (100)			0.2985 [a]
No		6 (17)	9 (27)	
Chemotherapy		6 (17)	7 (21)	
Radiotherapy		3 (9)	1 (3)	
Chemo and Radio		2 (6)	0 (0)	
**Relapse**	34 (100)			0.4646 [b]
No		7 (21)	4 (11)	
Yes		10 (30)	13 (38)	
**Mitotic count (mitoses/10 high-power fields)**	20 (100)			1.000 [b]
≤10		1 (5)	2 (10)	
≥10		6 (30)	11 (55)	
**Metastasis**	34 (100)			0.4848 [b]
No		2 (6)	0 (0)	
Yes		15 (44)	17 (50)	
**Metastasis Localization**	23 (100)			0.0743 [b]
Local (pelvic)		1 (5)	7 (30)	
Distant		9 (39)	6 (26)	

[a] Chi-square; [b] Fisher exact; FIGO, International Federation of Gynecology and Obstetrics.

Table S7. Comparison between clinical and pathological features and the *let-7e* expression.

Variables	n (%)	*let-7e*		*p*
		Downregulated	Upregulated	
**Age (years)**				
<50	**34 (100)**	5 (15)	9 (26)	0.2960[b]
≥50		12 (35)	8 (24)	
**Menopause**	31 (100)			
No		5 (16)	3 (10)	0.4331[b]
Yes		10 (32)	13 (42)	
**Histologic grade**	34 (100)			
Low grade		3 (9)	3 (9)	1.000[b]
High grade		14 (41)	14 (41)	
**Clinical FIGO stage**	34 (100)			0.2930[a]
I		9 (26)	4 (12)	
II		2 (6)	3 (9)	
III		3 (9)	3 (9)	
IV		3 (9)	7 (20)	
**Adjuvant therapy**	34 (100)			0.2887[a]
No		7 (21)	8 (23)	
Chemotherapy		5 (15)	8 (23)	
Radiotherapy		3 (9)	1 (3)	
Chemo and Radio		2 (6)	0 (0)	
**Relapse**	34 (100)			0.7139[a]
No		5 (15)	6 (18)	
Yes		12 (35)	11 (32)	
**Mitotic count (mitoses/10 high-power fields)**	20 (100)			1.000[b]
≤10		1 (5)	2 (10)	
≥10		6 (30)	11 (55)	
**Metastasis**	34 (100)			1.000[b]
No		1 (3)	1 (3)	
Yes		16 (47)	16 (47)	
**Metastasis Localization**	23 (100)			**0.0094[b]**
Local (pelvic)		1 (5)	7 (30)	
Distant		11 (48)	4 (17)	

[a] Chi-square; [b] Fisher exact; FIGO, International Federation of Gynecology and Obstetrics.

Table S8. Comparison between clinical and pathological features and the *let-7f* expression.

Variables	n (%)	*let-7f*		*p*
		Downregulated	Upregulated	
**Age (years)**				
<50	**34 (100)**	5 (15)	9 (26,5)	0.3151
≥50		11 (32)	9 (26,5)	
**Menopause**	31 (100)			
No		5 (16)	3 (10)	0.4125 [b]
Yes		9 (29)	14 (45)	
**Histologic grade**	34 (100)			
Low grade		4 (12)	2 (6)	0.3872 [b]
High grade		12 (35)	16 (47)	
**Clinical FIGO stage**	34 (100)			0.2223 [a]
I		8 (23)	5 (15)	
II		3 (9)	2 (6)	
III		3 (9)	3 (9)	
IV		2 (6)	8 (23)	
**Adjuvant therapy**	34 (100)			0.6488 [a]
No		7 (21)	8 (23)	
Chemotherapy		5 (15)	8 (23)	
Radiotherapy		3 (9)	1 (3)	
Chemo and Radio		1 (3)	1 (3)	
**Relapse**	34 (100)			0.7166 [a]
No		6 (17)	5 (15)	
Yes		10 (30)	13 (38)	
**Mitotic count (mitoses/10 high-power fields)**	20 (100)			1.000 [b]
≤10		1 (5)	2 (10)	
≥10		6 (30)	11 (55)	
**Metastasis**	34 (100)			0.2139 [b]
No		2 (6)	0 (0)	
Yes		14 (41)	18 (53)	
**Metastasis Localization**	23 (100)			0.3788 [b]
Local (pelvic)		2 (9)	6 (26)	
Distant		8 (35)	7 (30)	

[a] Chi-square; [b] Fisher exact.

Table S9. Comparison between clinical and pathological features and the *let-7g* expression.

Variables	n (%)	*let-7g*		*p*
		Downregulated	Upregulated	
**Age (years)**				
<50	**34 (100)**	8 (24)	6 (18)	0.4858 [a]
≥50		9 (26)	11 (32)	
**Menopause**	31 (100)			
No		3 (10)	5 (16)	0.6980 [b]
Yes		11 (36)	12 (38)	
**Histologic grade**	34 (100)			
Low grade		3 (9)	3 (9)	1.000 [b]
High grade		14 (41)	14 (41)	
**Clinical FIGO stage**	34 (100)			0.1584 [a]
I		9 (27)	4 (12)	
II		3 (9)	2 (6)	
III		1 (3)	5 (15)	
IV		4 (11)	6 (17)	
**Adjuvant therapy**	34 (100)			0.6239 [a]
No		8 (23)	7 (21)	
Chemotherapy		5 (15)	8 (23)	
Radiotherapy		3 (9)	1 (3)	
Chemo and Radio		1 (3)	1 (3)	
**Relapse**	34 (100)			0.4646 [b]
No		7 (21)	4 (12)	
Yes		10 (29)	13 (38)	
**Mitotic count (mitoses/10 high-power fields)**	20 (100)			1.000 [b]
≤10		1 (5)	2 (10)	
≥10		7 (35)	10 (50)	
**Metastasis**	34 (100)			0.4848 [b]
No		2 (6)	0 (0)	
Yes		15 (44)	17 (50)	
**Metastasis Localization**	23 (100)			0.3788 [b]
Local (pelvic)		2 (9)	6 (26)	
Distant		8 (35)	7 (30)	

[a] Chi-square; [b] Fisher exact; FIGO, International Federation of Gynecology and Obstetrics.

Table S10. Comparison between clinical and pathological features and the *let-7i* expression.

Variables	n (%)	*let-7i*		*p*
		Downregulated	Upregulated	
**Age (years)**				
<50	**34 (100)**	8 (24)	6 (18)	0.4858 [a]
≥50		9 (26)	11 (32)	
**Menopause**	31 (100)			
No		3 (10)	5 (16)	0.6980 [b]
Yes		11 (36)	12 (38)	
**Histologic grade**	34 (100)			
Low grade		2 (6)	4 (12)	0.6562 [b]
High grade		15 (44)	13 (38)	
**Clinical FIGO stage**	34 (100)			0.1584 [a]
I		9 (27)	4 (12)	
II		3 (9)	2 (6)	
III		1 (3)	5 (15)	
IV		4 (11)	6 (17)	
**Adjuvant therapy**	34 (100)			0.9861 [a]
No		8 (23)	7 (21)	
Chemotherapy		6 (17)	7 (21)	
Radiotherapy		2 (6)	2 (6)	
Chemo and Radio		1 (3)	1 (3)	
**Relapse**	34 (100)			1.000 [a]
No		6 (17)	5 (15)	
Yes		11 (33)	12 (35)	
**Mitotic count (mitoses/10 high-power fields)**	20 (100)			0.5211 [b]
≤10		0 (0)	3 (15)	
≥10		7 (35)	10 (50)	
**Metastasis**	34 (100)			0.4848 [b]
No		2 (6)	0 (0)	
Yes		15 (44)	17 (50)	
**Metastasis Localization**	23 (100)			0.1930 [b]
Local (pelvic)		2 (9)	6 (26)	
Distant		9 (39)	6 (26)	

[a] Chi-square; [b] Fisher exact; FIGO, International Federation of Gynecology and Obstetrics.
